# Selective Pulmonary Artery Perfusion With Blood Flow Occlusion Delivers Concentrated Levels of Chemotherapy to Ipsilateral Hilar and Mediastinal Lymph Nodes

**DOI:** 10.14740/wjon788w

**Published:** 2014-03-11

**Authors:** Preston J. Sparks, Jesse Hines, Jake Lowry, Matthew Strode, Michael Roach

**Affiliations:** a General Surgery, Dwight David Eisenhower Army Medical Center, Fort Gordon, GA, USA; b PerkinElmer, Johns Creek, GA, USA; c Department of Clinical Investigations, Dwight David Eisenhower Army Medical Center, Fort Gordon, GA, USA; d Cardiothoracic Surgeon, Dwight David Eisenhower Army Medical Center, Fort Gordon, GA, USA

**Keywords:** Selective pulmonary artery perfusion, Regional chemotherapy, Mediastinal lymph nodes

## Abstract

**Background:**

Although survival is historically low for patients presenting with N2 lung cancer, patients who respond to chemotherapy have up to a 30% chance for long term survival or cure. Selective pulmonary artery perfusion (SPAP) has been examined in several animal studies as a method for delivering chemotherapy in non-small cell lung cancer; however, there is a paucity of data regarding the effect of SPAP on regional lymph nodes.

**Methods:**

Left SPAP was performed using gemcitabine on five swine and compared with standard central venous infusion in controls. Samples were taken from lung, kidney, liver, plasma, and lymph nodes. Tissue was measured for gemcitabine concentration using mass spectroscopy.

**Results:**

Left SPAP resulted in significantly higher gemcitabine concentration than standard infusion in hilar and mediastinal lymph nodes while plasma gemcitabine concentration was not significantly different.

**Conclusion:**

SPAP is a viable technique for concentrating a chemotherapeutic agent in the mediastinal and hilar lymph nodes. This could potentially increase the response to chemotherapy and render more patients to be surgical candidates who present with N2 disease.

## Introduction

Lung cancer is the leading cancer killer in the United States. Only 15% of patients have disease confined to the lung at diagnosis. While these patients clearly benefit from surgery, there is another 22% of patients who have regional nodes involved at the time of diagnosis who may or may not benefit from surgery based on the extent of disease and response to chemotherapy [1, 2]. The primary factor determining which patients will benefit from surgery is the presence or absence of mediastinal lymph node involvement. Historically, randomized controlled trials have failed to show a benefit to surgery over definitive chemo-radiation for patients with mediastinal lymph node involvement (N2 disease) [3, 4]. Recent studies evaluating neo-adjuvant chemotherapy in patients with N2 disease have demonstrated that roughly half of the patients will have some degree of pathological response to induction chemotherapy and a proportion of those will have pathologic down staging to N1 or N0 disease. Of the patients who respond to induction chemotherapy and undergo surgery, the 5-year survival may be as high as 30% which is a remarkable improvement over a long term survival of 7-16% without surgery [5, 6]. Selective pulmonary artery perfusion (SPAP) is a method of local chemotherapy delivery whereby either the left or right pulmonary artery is accessed and direct chemotherapy infusion is performed. This study was performed to assess whether SPAP would result in the concentrated delivery of gemcitabine to the regional lymph nodes. In this study, we performed left SPAP on five swine with gemcitabine and measured the tissue concentration of the drug in both the hilar and mediastinal lymph nodes. We compared these results to controls that received standard central venous chemotherapy infusion (CVI).

## Materials and Methods

### Animals

Ten female Yorkshire pigs weighing 65 - 75 kg were used under approved animal use protocol in compliance with the local IACUC. Animals were fed and housed in accordance with the Animal Welfare Act. All animals were sacrificed at the end of the procedure under general anesthesia.

### Anesthesia and euthanasia

Pigs were anesthetized with an induction agent combination of xylazine (1 mg/kg) and telazol (5.5 mg/kg) intramuscularly and an auricular vein was catheterized for IV fluid administration at 5 - 10 mL/kg/h. Atropine was also given at a dose of 0.05 mg/kg IM for secretions. Animals were intubated and maintained under general anesthesia using a combination of isoflurane gas (MAC 0.5-4.0%) with a continuous infusion of fentanyl (50 - 100 µg/kg/h). Animals were ventilated at a rate of 10 - 14 per minute. During the dissection of the left pulmonary artery and the mediastinal lymph node dissection, an infusion of amiodarone (10 mg/kg/h) and lidocaine (50 µg/kg/min) was required to prevent dysrhythmias. Dopamine (2 - 20 µg/kg/min) was also started to maintain blood pressure. Animals were monitored for heart rate, rhythm, arterial blood pressure, end tidal CO_2_, and oxygen saturation. At the completion of the procedure, the animal was euthanized under general anesthesia.

### Surgical technique

**Figure 1:**
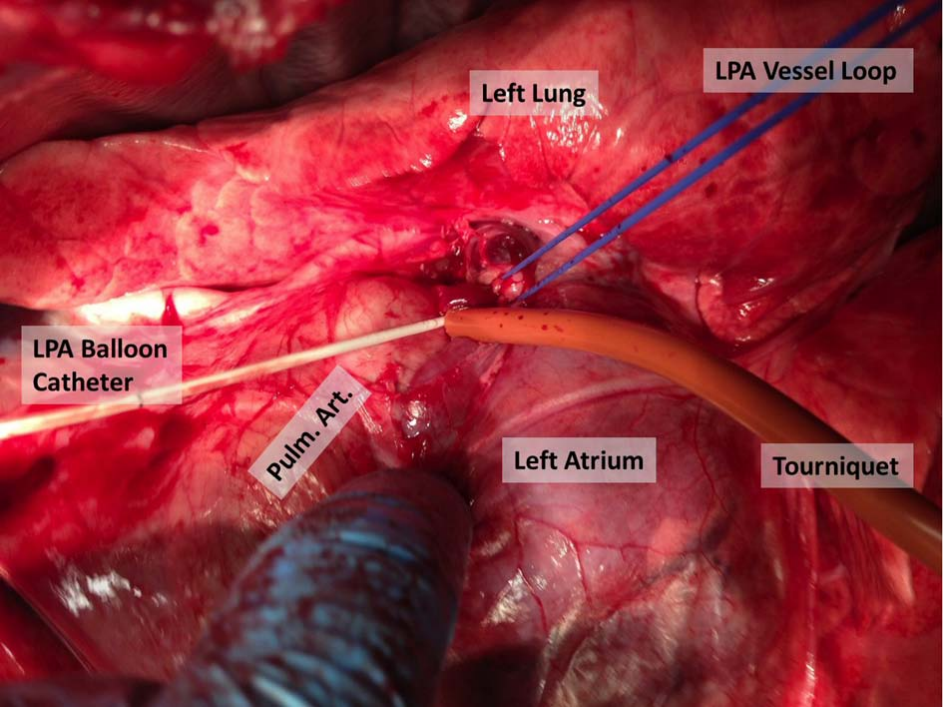
Technique for left pulmonary artery isolation and occlusion.

CVI was performed by right internal jugular vein cutdown and catheterization using a 16-gauge 8 cm catheter. SPAP was performed by midline sternotomy. First, the intra-pericardial left pulmonary artery was isolated and encircled with a vessel loop (Fig. 1). An arteriotomy was created in the main pulmonary artery and a 6F Fogarty 13 mm ballon catheter was introduced through the arteriotomy and directed by hand into the left pulmonary artery. The balloon was inflated in the left pulmonary artery and the vessel loop was tightened prior to retracting the catheter proximally to the vessel loop ensuring occlusion of the left pulmonary artery. The intra-pericardial pulmonary artery was accessed to minimize the potential for lymphatic disruption during dissection. Hemostasis was obtained on the arteriotomy by using a purse string suture with 5-0 prolene in a Rummel tourniquet. Chemotherapy infusion was performed over 30 min using a standard intravenous infusion pump for both infusion techniques.

Following chemotherapy infusion, the balloon catheter was removed and blood flow to the left pulmonary artery was restored. The arteriotomy was closed with the purse string suture. A bilateral mediastinal lymph node dissection was performed and anterior and posterior lung samples were taken from each lobe. Tissue samples were also taken from the liver and kidney. Plasma was drawn from the right atrium following chemotherapy infusion.

### Gemcitabine

All animals received gemcitabine at 1,250 mg/m^2^ body surface area which was calculated using the formula BSA = [weight (kg) × 0.0734]^0.656^. Gemcitabine was delivered in 100 mL normal saline over 30 min.

### Tissue processing and analysis

All samples were immediately frozen under liquid nitrogen after collection and stored at -80 °F until processing. Tissue samples were homogenized under liquid nitrogen. Between 50 and 100 mg of frozen homogenized tissue was weighed and 99% acetonitrile was added at a volume of 10 µL/mg tissue. The acetonitrile contained an internal standard of gemcitabine ^13^C_2_ at a volume of 1.4 µg/mL. Extraction was then performed by briefly vortexing the samples and the samples were placed on a rocker for 10 min. The samples were then centrifuged at 10,000 rpm for 90 s and the supernate was collected and frozen for processing by mass spectroscopy.

Mass spectroscopy was performed using a PerkinElmer (Johns Creek, GA, USA) time of flight mass spectrometer and direct sample analyzer (DSA). Each sample was run in triplicate. Spectra were analyzed by summing the area under each peak. Tissue concentration was calculated by dividing the gemcitabine area by the internal standard. Further details regarding this method will be published elsewhere.

### Statistics and data analysis

The concentration of gemcitabine in each sample was determined be the mean ratio of the three sample runs between the drug and internal. Significance testing was performed using an unpaired Student’s t test. Statistical significance was defined as a P value ≤ 0.05.

## Results

Left SPAP resulted in significantly higher gemcitabine concentrations than CVI in the left hilar lymph nodes (52.30 ± 13.34 vs. 1.51 ± 0.13, P = 0.01) (Table 1). The gemcitabine concentration in the level 5 mediastinal nodes was higher in the SPAP group but this did not reach significance due to variability in the SPAP group (33.52 ± 15.67 vs. 1.54 ± 0.40, P = 0.06). In the level 7 lymph node the gemcitabine concentration was significantly higher in the SPAP group than in the CVI group (14.74 ± 4.91 vs. 1.35 ± 0.32, P = 0.02). In the right mediastinal lymph nodes (4R), there was no significant difference in gemcitabine concentration between the two infusion groups.

Comparing the left to the right sided lymph node concentrations in the SPAP group shows that there was significantly higher gemcitabine concentrations in the left versus the right sided nodes. Mediastinal lymph node gemcitabine concentrations were significantly higher on the left compared to the right (33.52 ± 15.67 vs. 2.33 ± 0.41, P = 0.04). The gemcitabine concentrations in hilar lymph node were also significantly higher on the left compared to the right (52.30 ± 13.34 vs. 2.54 ± 0.18, P < 0.01).

**Figure 2:**
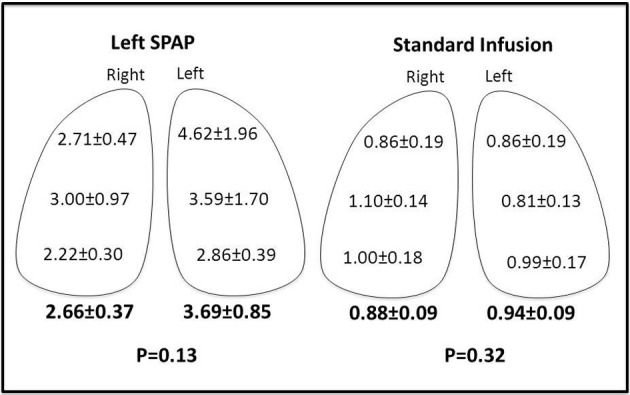
Lung concentrations of gemcitabine (µg/g tissue).

In the SPAP group, lung tissue gemcitabine concentrations were significantly higher than in the CVI group in both the right and left lung. In the Left SPAP group, gemcitabine levels were higher in the left lung versus the right lung but this was not significant due to sample variability. Lung levels were essentially the same bilaterally with standard chemotherapy infusion (Fig. 2).

Plasma, kidney, and liver levels were higher in the SPAP group although this was only significant in the liver (Table 1).

## Discussion

Several studies have examined the effect of SPAP on the lung tissue, and there are very little data regarding what effect this has on the lymph nodes. In 2009, Demmy et al published preliminary results of an isolated lung suffusion trial in humans [7]. He mentions that in his canine model, lymphoid atrophy of the tracheobronchial nodes was seen in the surviving animals but no quantitation of the drugs was reported. Also in 2009, Van Putt et al reported that in the level 5 lymph node concentrations of gemcitabine and carboplatin in their results with SPAP in pigs [8]. Their technique differed from ours in that they introduced the balloon catheter through the internal jugular vein and used the waveforms to guide placement while we placed our catheter surgically. They also infused gemcitabine over only 2 min without PA occlusion and then occluded the PA for 30 min following the infusion while we occluded the PA first and infused gemcitabine over 30 min. They report no significant difference in the lymph node concentrations of gemcitabine between SPAP and CVI.

In our study, we demonstrated markedly elevated hilar and mediastinal lymph node concentrations of gemcitabine with SPAP compared to CVI on the order of 34 and 21 fold, respectively. Level 5 lymph node concentrations were variable in the SPAP group (4.63 - 62.99 µg/g) and thus did not reach significance (P = 0.057). This was possibly due to the dissection performed to control the left pulmonary artery interfering with lymphatics and sampling error due to a large level 5 lymph node packet present in swine. Level 7 lymph nodes were significantly higher with SPAP and less variable (3.60 - 23.46 µg/g).

Additionally, SPAP achieved ipsilateral lymph node drug concentrations that were 20 and 14 fold higher in the hilar and mediastinal nodes, respectively compared to the contralateral side. The reason that our results differed from those of Van Putt is likely the fact that blood flow remains intact from the bronchial artery and there will inevitably be washout of the drug even with blood flow occlusion. Therefore, a slow infusion during blood flow occlusion will deliver a concentrated dose over the entire time period of balloon occlusion.

Lung concentrations of gemcitabine were higher on the left than right in the SPAP group (2.66 vs. 3.69); however, this was not statistically significant due to high sample variability. This is consistent with a previous study which demonstrated heterogeneous drug distribution with isolated lung perfusion as compared to CVI [9]. Our study showed very little variability in the gemcitabine concentration in the CVI group.

**Figure 3:**
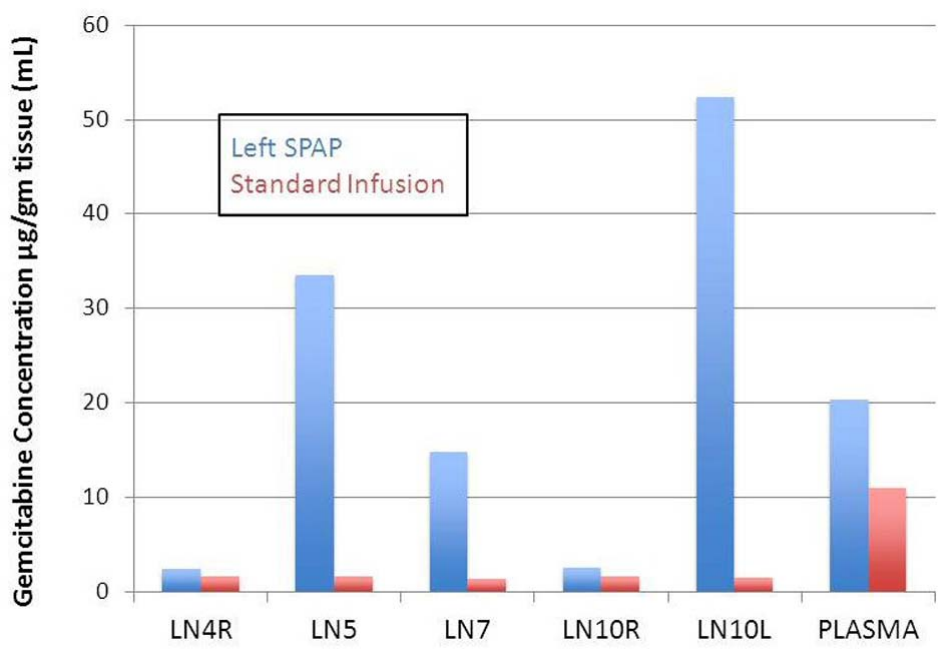
Comparison of gemcitabine concentration in lymph nodes and plasma for left SPAP and standard chemotherapy infusion.

Plasma, liver, and kidney concentrations of gemcitabine were higher in the SPAP group compared with CVI although this only reached significance in the liver (Fig. 3). Plasma levels in our study ranged from 10.40 to 42.42 µg/mL (mean 20.34) in the SPAP group which were higher than CVI which ranged 7.24 - 15.73 µg/mL (mean 10.96). Both groups received the same dose of chemotherapy over 30 min. The higher levels seen in the SPAP group likely represent the rapid washout of gemcitabine from the pulmonary vascular bed after restoration of blood flow to the left pulmonary artery. These results are consistent with Van Putt’s report in which lung and serum levels were initially higher in the SPAP group but were similar to the CVI group at 30 and 20 min time points, respectively. We expect that if we had assessed plasma levels at a later time point, we would see similar results.

This small study by no means answers all the questions that need to be addressed before a phase I trial of SPAP could be conducted. Further studies need to be done to demonstrate these same results can be achieved percutaneously as median sternotomy may change the physiology of the chest lymphatics and dissection around the left pulmonary artery may potentially alter lymphatic drainage to the level 5 lymph nodes. Furthermore, the optimal drug and dose still needs to be determined. What these results do show is that the pathways exist to deliver a concentrated dose of chemotherapy to both N1 and N2 lymph nodes using SPAP with blood flow occlusion. This could potentially improve response rates to induction chemotherapy, making more patients with node positive lung cancer candidates for surgery with the potential for improved long term survival.
